# Harnessing DNA-Based Technology for Drug Discovery

**DOI:** 10.1371/journal.pbio.0020222

**Published:** 2004-06-22

Traditionally, developing small molecules for research or drug treatments has been a painstaking enterprise. Drugs work largely by binding to a target protein and modifying or inhibiting its activity, but discovering the rare compound that hits a particular protein is like, well, finding a needle in a haystack. With a specific protein target identified, scientists typically either gather compounds from nature or synthesize artificial compounds, then test them to see whether they act on the target.[Fig pbio-0020222-g001]

**Figure pbio-0020222-g001:**
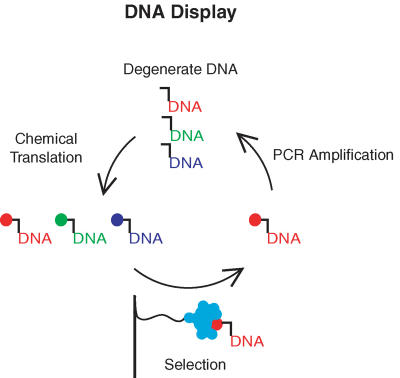
Small-molecule evolution

The birth of combinatorial chemistry in the early nineties promised to revolutionize this laborious process by offering a way to synthesize trillions of compounds at a time. Though molecules still had to be evaluated one by one, high-throughput screening technology could manage up to a million molecules a day. Despite these technological advances, few drugs have emerged from combinatorial chemistry approaches, leaving the promise largely unfulfilled.

Another strategy looks to nature as a model. The immune system fights disease and infection by generating billions of antibodies, each primed to recognize a specific pathogen. Antibodies recognize antigens (protein fragments of pathogens) with an exacting specificity that develops through an iterative process. The body first produces a diverse, random collection of antibodies. Every antibody is encoded with a unique DNA blueprint in a B-cell. Antibodies that happen to bind to a pathogen are “selected” to pass on their blueprints: successful binding stimulates cell division, during which blueprints are copied, varied by mutation, and used to create a new generation of antibodies. Specificity is refined over multiple generations.

Over the past fifteen years, biologists have developed techniques to recreate this process in a test tube. Today, it's common practice to “evolve” collections of as many as a quadrillion different proteins or nucleic acids to bind a molecular target. These techniques are called molecular breeding, because like traditional livestock and crop breeding techniques, they combine sets of genotypes over generations to produce a desired phenotype. Molecular breeding has so far been restricted only to applications that involve materials encoded by DNA. Drugs produced by conventional synthetic organic chemistry, for example, cannot be bred.

In a series of three articles in this issue of *PLoS Biology*, David Halpin et al. describe a strategy that addresses this limitation. By inventing a genetic code that acts as a blueprint for synthetic molecules, the authors show how chemical collections of nonbiological origin can be evolved. In the first article, Halpin et al. present a method for overcoming the technical challenge of using DNA to direct the chemical assembly of molecules. In the second, they demonstrate how the method works and test its efficacy by creating a synthetic library of peptides (protein fragments) and then showing that they can find the “peptide in a haystack” by identifying a molecule known to bind a particular antibody. The third paper shows how the method can support a variety of chemistry applications that could potentially synthesize all sorts of nonbiological “species.” Such compounds, the authors point out, can be used for drug discovery or as molecular tools that offer researchers novel ways to disrupt cellular processes and open new windows into cell biology. While medicine has long had to cope with the evolution of drug-resistant pathogens, it may now be possible to fight fire with fire. To learn more about the DNA display method described here, see the primer “Translating DNA into Synthetic Molecules,” also in this issue.

